# 

**Published:** 1877-09

**Authors:** 


VOL. 24. SEPTEMBER, 1877. No. 9.

TWENTY-FOURTH YEAR.

### HALL'S Journal of Health.

PUBLISHED AT BIBLE HOUSE,
THIRD AVENUE, CORNER OF NINTH STREET,
NEW YORK.

E. H. GIBBS, M.D., EDITOR.

AGENTS FOR THE TRADE:
AMERICAN NEWS COMPANY, 121 NASSAU STREET.
NEW YORK NEWS COMPANY, 18 BEEKMAN STREET.

**Terms, $1.50 a Year.**
**SINGLE NUMBER, 15 CENTS.**

KENT & CO., Printers, 11 Frankfort St., New York.

